# A rapid evaluation method of blasting effect based on optimized image segmentation algorithm and application in engineering

**DOI:** 10.1038/s41598-024-55369-y

**Published:** 2024-02-27

**Authors:** Peng He, Yifan Xu, Feng Jiang, Gang Wang, Zhiyong Xiao, Chengcheng Zheng

**Affiliations:** 1 Shandong University of Science and Technology, Qingdao, 266590 China; 2 College of Civil Engineering, Fujian University of Technology, Fuzhou, 350118 China

**Keywords:** Blasting blocks, Image acquisition, Improved image segmentation algorithm, Self-developed software, Engineering site, Civil engineering, Energy infrastructure

## Abstract

To quickly determine the blasting block degree and conduct an accurate and objective analysis of the tunnel blasting effect, this study has enhanced and improved upon the traditional genetic algorithm and Otsu algorithm. It has combined it with the marking watershed method and utilized ground digital acquisition to capture images of blasting debris. These images are then used in our custom-developed blasting analysis software to calculate the blasting block degree distribution and provide a quantitative analysis of blasting block degree. The research results show that the optimized image segmentation algorithm effectively improves the traditional threshold segmentation method on the poor effect of segmentation of the edge of the adherent block or the direct application of the watershed segmentation of the over-segmentation problem, to improve the segmentation accuracy based on the new segmentation technology is close to the traditional technology in terms of time. Through the self-developed software, the construction personnel in the project site to quickly obtain the blasting block degree histogram, block degree cumulative curve and other important indicators of the evaluation of the effect of blasting block degree, to provide data support for on-site construction, to assist in the modification of the blasting program, and to improve the efficiency of construction. This study realizes the rapid detection and block identification of blasting blocks, provides data support for the optimization of blasting parameters, and has good application and promotion value.

## Introduction

With the growing demand for transportation, highways, passenger and freight railways, as well as urban underground railways, are rapidly expanding and being constructed. Drilling and blasting method has become a prevalent method of tunnelling due to its mature technology, simple construction machinery, and low costs. However, if the geological conditions of the surrounding rock are not accurately analyzed during the drill and blast method, it can lead to poor blasting results, resulting in irregular tunnel contours, blasting roots, and blasting chunks. Blasting chunks are the most common blasting defect, and an increase in their rate can significantly impact shovel loading efficiency and construction safety. Therefore, a rapid and accurate evaluation of blasting effect is crucial for developing an excavation plan, determining equipment and processes.

In order to evaluate the blasting effect of the drilling and blasting method, scholars have carried out a lot of research from theoretical analysis, experimental research and numerical simulation. The blasting effect of the drill-and-blast method is comprehensively judged by primary indicators such as safety, quality and economic benefits in the blasting process and secondary indicators such as blasting block size^1,2^. Due to the excessive number of indicators in the judgment evaluation system of the first-level indicators, it is too complex and difficult to quantify and normalise, resulting in inaccurate and non-objective evaluation results. Most of the research direction focuses on favouring the analysis of blasting parameters and blasting chunks. The study of blasting parameters can be analysed by numerical simulation and field test^3^, in which case the blasting parameters may not be able to make a quick evaluation of the field situation, so the study should focus on the quick acquisition of the distribution of the blasted chunks to evaluate the blasting effect.

Based on the research of many scholars in the past, it is known that the distribution of rock mass degree of the blasting blocks can be scientifically and accurately evaluated for the blasting effect. Image processing technology can identify and extract the edges and contours of the rock after blasting, so as to get the shape and size information of the rock, we expect to start from the image segmentation processing technology, to reduce the workload and error of manual sieving and counting, and to process the captured image of blasting lumpiness, in order to realize the rapid acquisition of blasting lumpiness. A lot of technical research has been carried out by scholars through image recognition analysis. In the dim environment of tunnels, the computer vision recognition monitoring system is used to characterize the image using multiple image processing algorithms to extract the length and width of the target. Additionally, a human–computer interaction recognition system has been designed to improve recognition accuracy, accelerate recognition speed, and achieve real-time calibration^4–7^. Furthermore, a photogrammetric system based on image matching and other methods has been proposed for underground detection and prevention in tunnels^8^.

Numerous scholars’ research has proved that image recognition in dim tunnels is feasible. Various techniques have been attempted to determine blasting block size, including small-scale blasting screening methods, empirical formulas, computer simulations, and photographic methods. Modern techniques utilizing video images and computer image processing techniques have the potential to analyze rock fragmentation accurately and efficiently^9^. Most existing techniques have been used to obtain the distribution of fractured rock through the extraction of image texture features. Researchers have primarily utilized Gaussian mixture models^10^, Fourier transform coefficients, local binary modes, and Gabor filters^11^ to acquire size distribution data of fractured rocks. Hamzeloo^12^ has developed a machine learning based neural network model to segment and process fractured rock. However, due to uneven lighting, shadows, noise and fragment size in the tunnel, standard edge detection methods often fail to achieve accurate representation. In addition, when segmenting adhered blasting blocks, excessive surface noise can lead to insufficient segmentation, resulting in significant errors in quantitative results after image segmentation. Furthermore, some existing methods for blasting block size analysis are prone to problems such as recognition errors, omissions and irregular morphology of blasting blocks in complex scenes, which can affect the effectiveness and speed of image segmentation.

Numerous researchers have attempted to address the above problems using various algorithms. Ji^13^ used the ACE + CLAHE algorithm to process images of the size of blasting blocks in tunnels, enabling accurate identification of the size of blasting blocks despite uneven illumination. Purswani^14^ use fast random forest and clustering analysis of machine learning methods for segmentation of porous media image processing. Guo^15^ introduced the Phansalkar binarisation method and proposed a watershed seed point labelling method based on the solidity of the rock block contour, with the aim of establishing a fast and accurate method for rock debris particle size detection. A limiting factor in debris measurement techniques is their speed, as slower measurement processes can affect the overall mining operation^16^. Threshold segmentation, the most widely used image segmentation method, involves separating the target from the background by selecting an optimal threshold value, providing a basis for subsequent classification and recognition. However, selecting the optimal threshold to achieve the best segmentation effect has always been a challenge in threshold segmentation. Furthermore, the specialised nature of rock image analysis limits the availability of practitioners with both blasting expertise and software development capabilities, leaving users with fewer options for selecting mature blasting image processing software.

**Figure 1:**
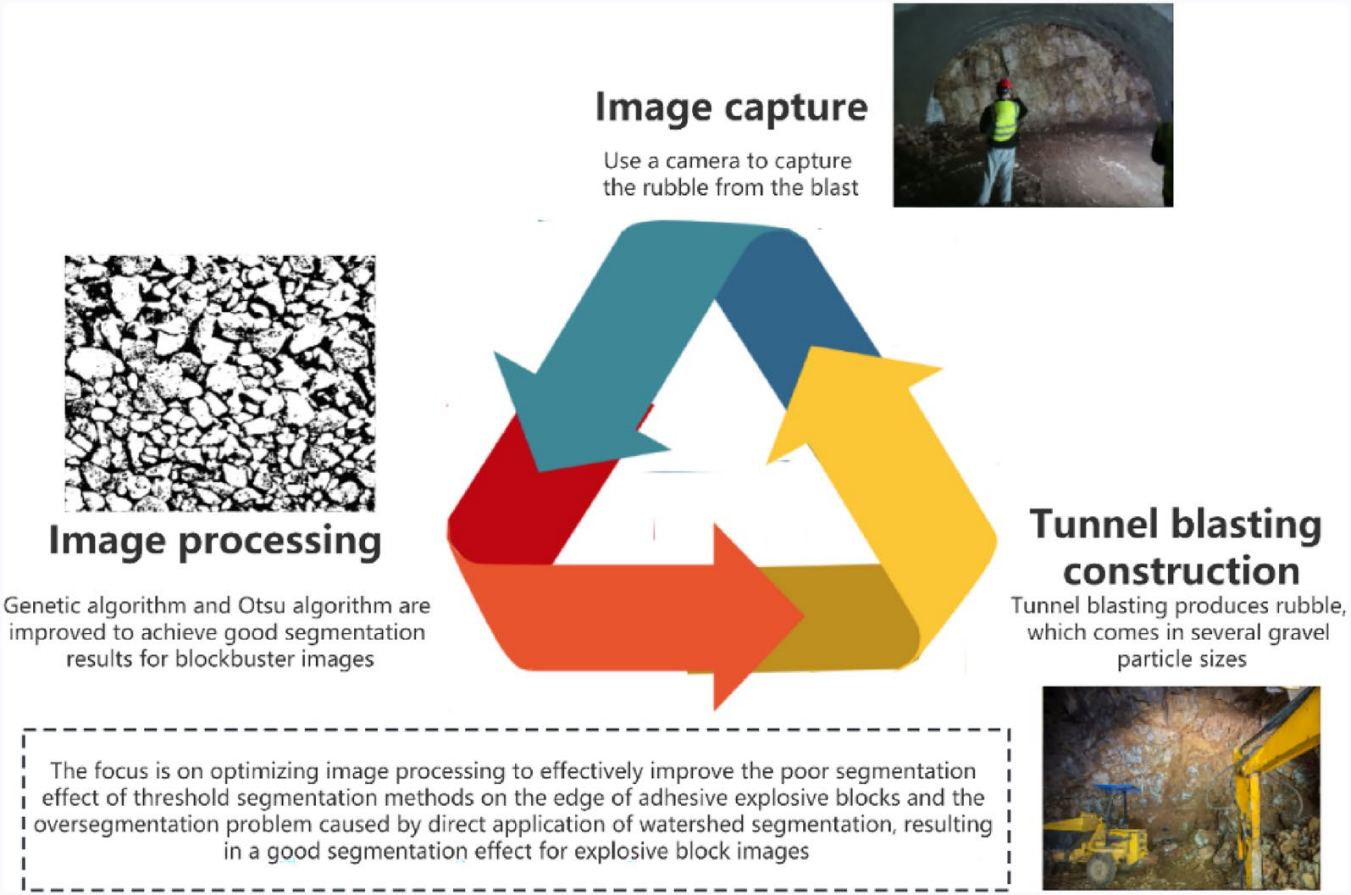
Graphical abstract.

In an effort to overcome the limitations of current blasting block size analysis methods, this paper introduces an improved genetic algorithm and the Otsu algorithm for pre-segmentation of blasting block images. This approach addresses the issue of over-segmentation in traditional threshold segmentation methods and enhances the edge segmentation of pixel blocks. Subsequently, the labeled watershed algorithm is utilized for secondary segmentation, enhancing segmentation accuracy without significantly increasing processing time. The development of a blasting effect analysis software enables the evaluation of blasting effectiveness based on key indicators such as the blasting blockiness histogram and lumpiness accumulation curve. This enables rapid detection and identification of blasting blocks, providing technical support for optimizing blasting parameters (Fig. 1). Ultimately, this approach aims to reduce construction costs and enhance blasting efficiency in engineering applications.

## Samples and equipment

**Figure 2:**
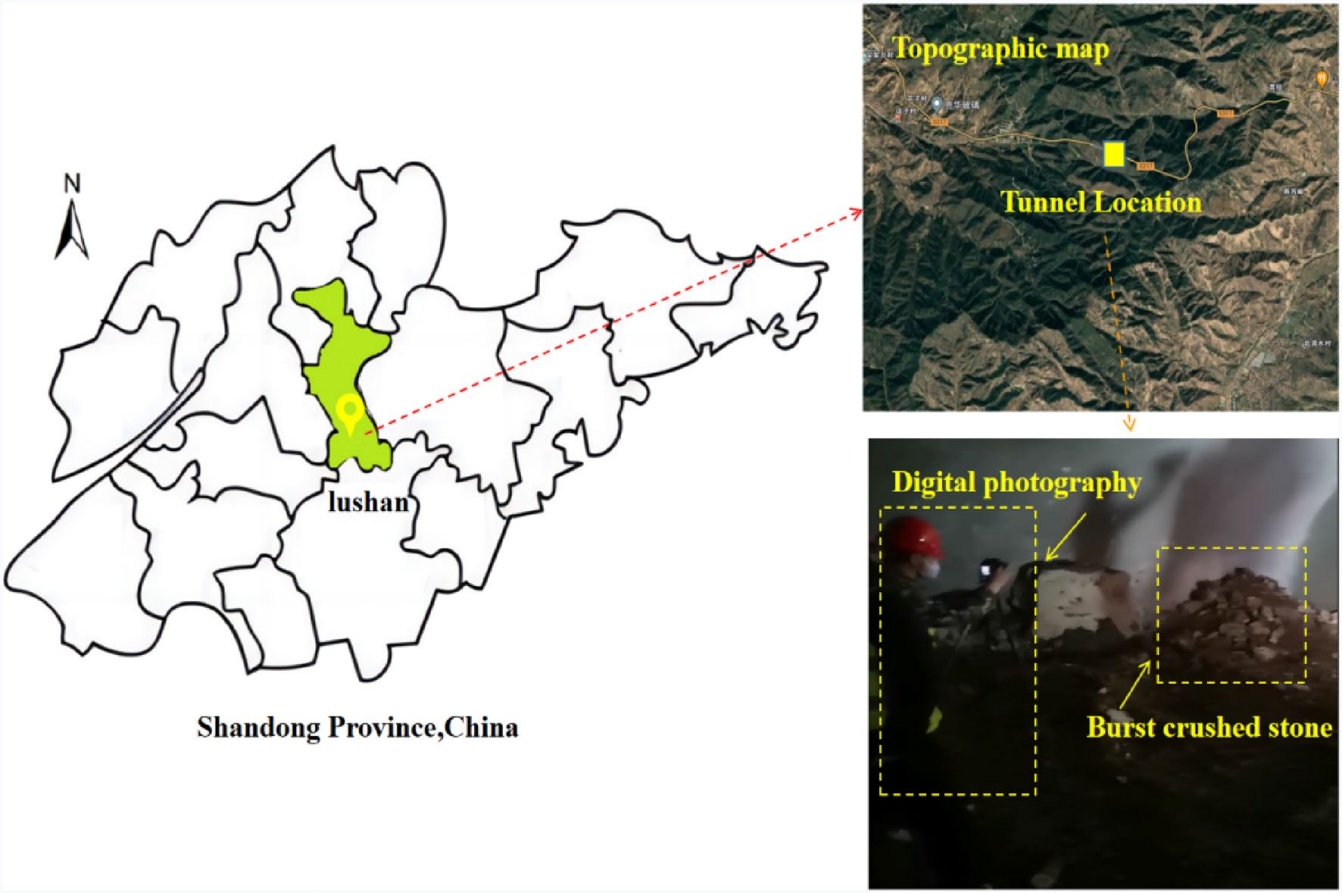
The location of Lushan Tunnel project and the use of digital photography to capture the blasting fragments inside the tunnel.

Blasting is a high effective method of tunnel construction in underground engineering. The purpose of blasting construction is to break the rock mass. If the size of the rock blocks after blasting does not meet the requirements, it will force the construction personnel to perform secondary crushing on the oversized blocks, which will affect the entire loading and transportation cycle, resulting in higher costs and lower tunneling efficiency. Blasting lumpiness refers to the geometric size of the broken rock mass after blasting, which is an important factor for evaluating the blasting effect. The rock particle size is classified as 0–0.5 m for fragments, 1–1.2 m or more for large blocks, and 1.2 m or more for blocks that need secondary crushing. The size of the lumpiness directly affects the subsequent loading and slagging efficiency of the broken rock mass. To facilitate transportation, rock blocks larger than 1.2 m may require mechanical crushing. Rapidly assessing blasting clumpiness is crucial for evaluating blasting effectiveness, enhancing blasting scheme design, and expediting construction progress. Figure 2 shows the site photo taken during the blasting construction of V-level surrounding rock in Lushan Tunnel.

**Figure 3:**
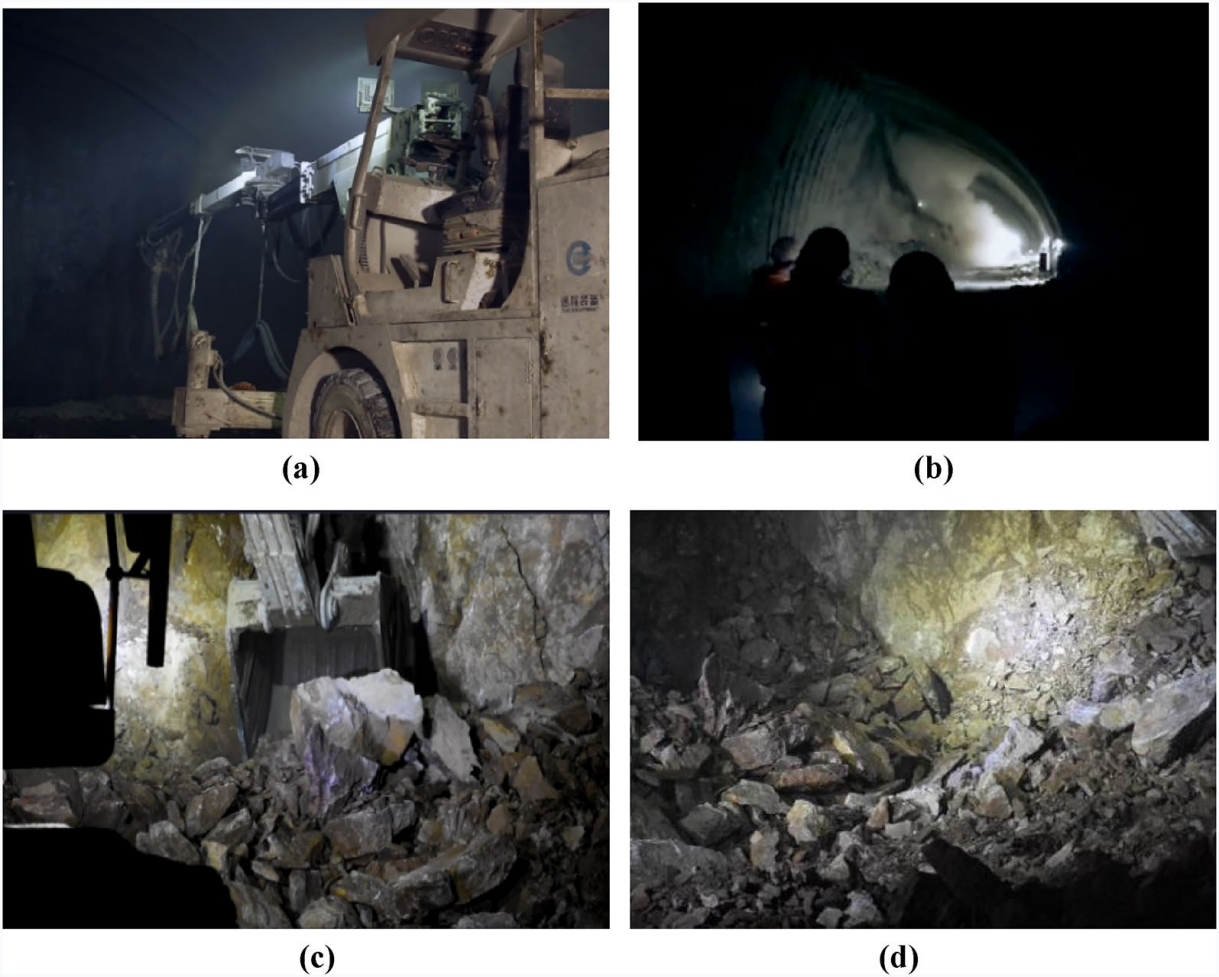
Collecting data on blasting lumpiness at the site. (**a**) Laying out gun holes. (**b**) Blasting site. (**c**) Scrapping. (**d**) Blasting rubble.

The process of tunnel blasting and excavation is shown in Fig. 3. Firstly, the tunnel palm face is drilled, as shown in Fig. 3a, emulsion explosives are placed in the holes, and then the explosives are ignited using a detonation controller to blow up the tunnel palm face, resulting in the blasting debris, the blasting site is shown in Fig. 3b, and the debris is dug out using an excavator, as shown in Fig. 3c, and the picture of the debris on the site after blasting is shown in Fig. 3d.

The existing methods for measuring the rock lumpiness after blasting can be summarized into two categories: traditional blasting lumpiness measurement methods and image processing-based blasting lumpiness measurement methods. The traditional objective lumpiness measurement methods, including the large block measurement method and sieving method, although the detection results are relatively accurate, have significant disadvantages such as large workload, high labor intensity of workers, small number of detection samples, and large interference to production, and can only be used to verify other methods. Traditionally, manual methods were used to manually depict the outline of the rock blocks on the blasting block photographs in the images and to calculate the number of squares of grid paper to be used in order to determine the block size. Nowadays, the computer-aided method of calculating the block size using the image recognition function saves a lot of time and provides useful and efficient data support for the project construction. The computer image processing-based method avoids the drawbacks of the traditional measurement method, and has become a research hotspot in the field of intelligent blasting and intelligent mine production in recent years.

**Figure 4:**
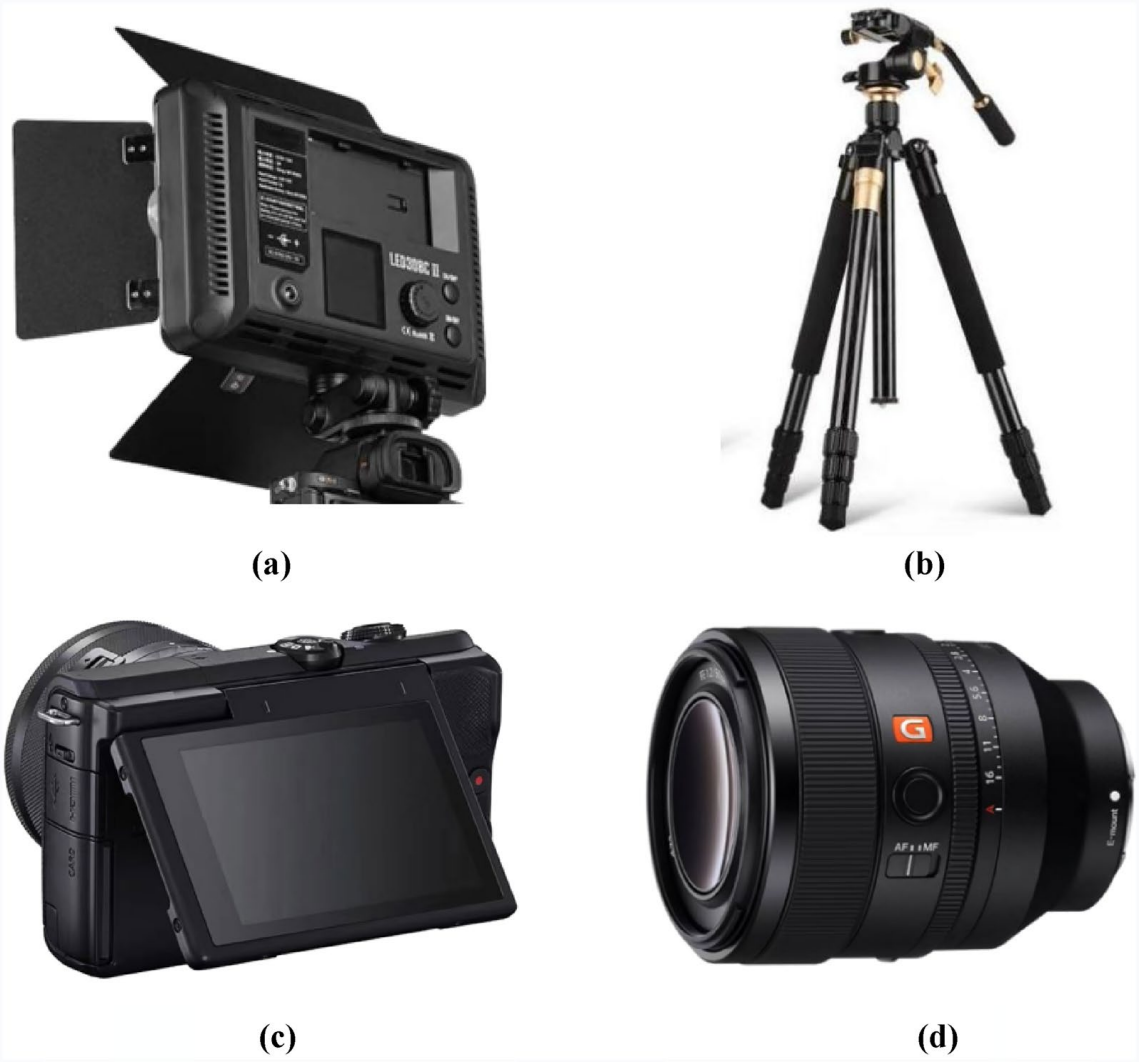
Equipment required for image acquisition. (**a**) Fill light. (**b**) Tripod. (**c**) Camera. (**d**) Lens.

This paper recommends using ground-based digital photography to extract images of blast fragments for accurate fragmentation measurement. As shown in Fig. 4. The fill light with 308 lamp beads, which restores the contour of the object itself more. The tripod used is Q999 SLR camera tripod, which can be matched with the fill light. The camera used has a maximum resolution of 4368 × 2912 with a maximum of 13.3 megapixels, a DIGIC II image processor, and a 2.5-in, 230,000-pixel LCD screen for clear image display. It utilizes a 35 mm full-frame CMOS image sensor that achieves high image quality with approximately 21.1 effective megapixels. The lens used offers excellent autofocus performance and portability.

**Figure 5:**
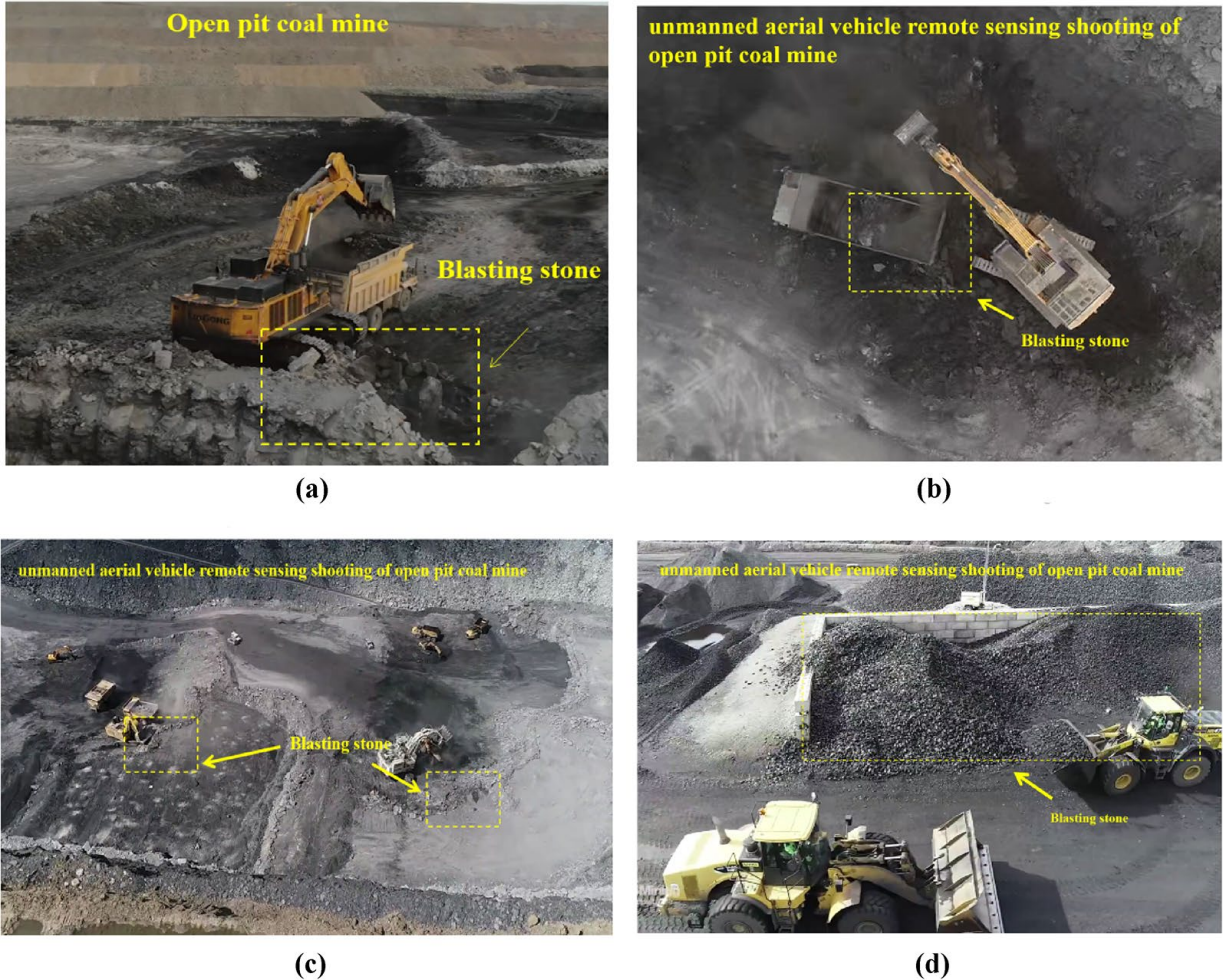
Schematic diagram of blasting rubble on site. (**a**) Schematic diagram of blasting rubble on site 1. (**b**) Schematic diagram of blasting rubble on site 2. (**c**) Schematic diagram of blasting rubble on site 3. (**d**) Schematic diagram of blasting rubble on site 4.

In order to show a more complete picture of the morphology of the blasting rags at the construction site, we used a drone to take pictures at the site. This is shown in Fig. 5.

**Figure 6:**
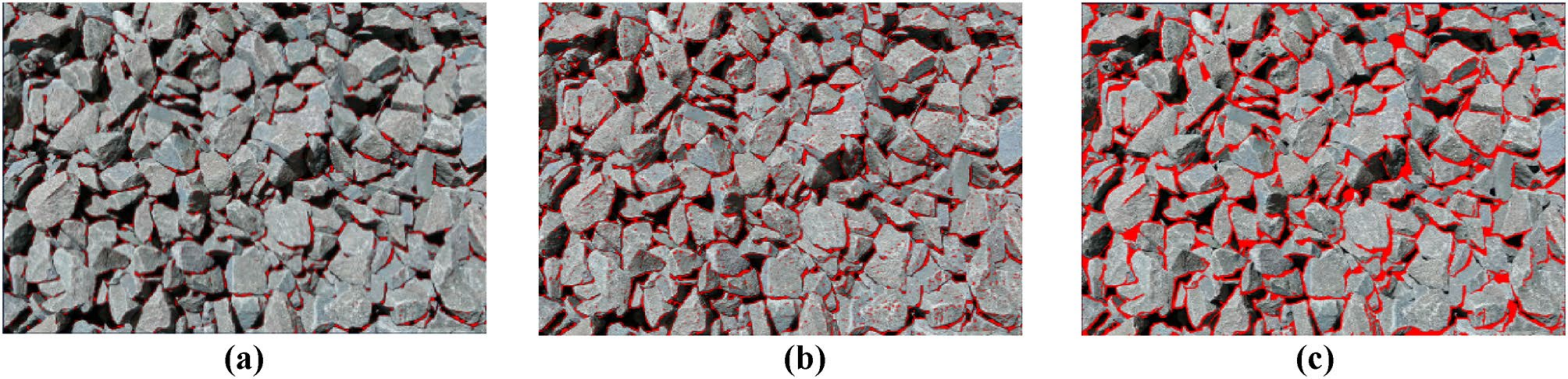
Broken stones photographed on site of Lushan tunnel. (**a**) Image recognition poor effect map. (**b**) Image recognition general effect picture. (**c**) Image recognition better effect picture.

Figure 6 shows an image of the blast heap, which was captured on-site during the surrounding rock blasting construction in Lushan Tunnel. The marked watershed algorithm was employed to segment blasting blocks, aiming to obtain the blasting block degree through machine vision method to guide construction. However, it was observed through practical application that even with clear blasting block images, the traditional threshold method often failed to achieve satisfactory image segmentation results due to issues such as variations in block size, stacking, complex textures, and similar gray values between the background and target. Therefore, exploring efficient and accurate image segmentation methods that minimize processing time and error is crucial for promptly assessing blasting block degree, evaluating blasting effectiveness, and facilitating prompt changes in blasting schemes.

## Proposed methods

**Figure 7:**
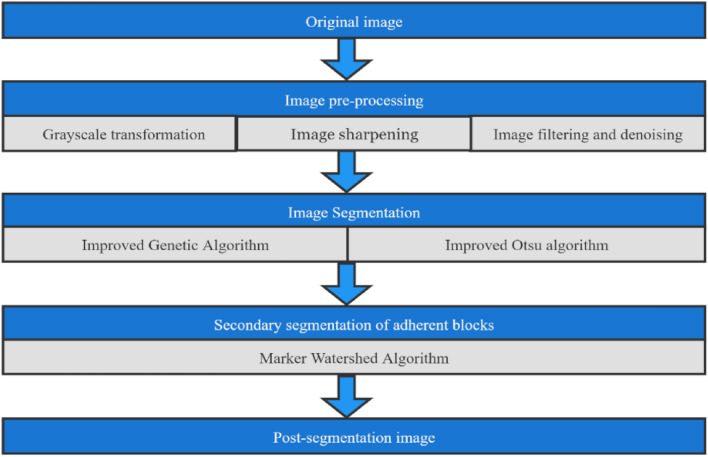
Research flow chart of this article.

In view of the problems existing in the calculation and analysis of blasting block degree in civil engineering construction, this study uses the improved genetic operator genetic algorithm and maximum inter-class variance method to pre-segment the blast heap, and then uses the marker watershed segmentation algorithm to perform secondary segmentation on the segmented binary image, in order to improve the poor segmentation effect of traditional threshold segmentation method on sticky blasting blocks, and control the increase of time consumption within an acceptable range. The specific flow chart is shown in Fig. 7.

### Blasting block image preprocessing

**Figure 8:**
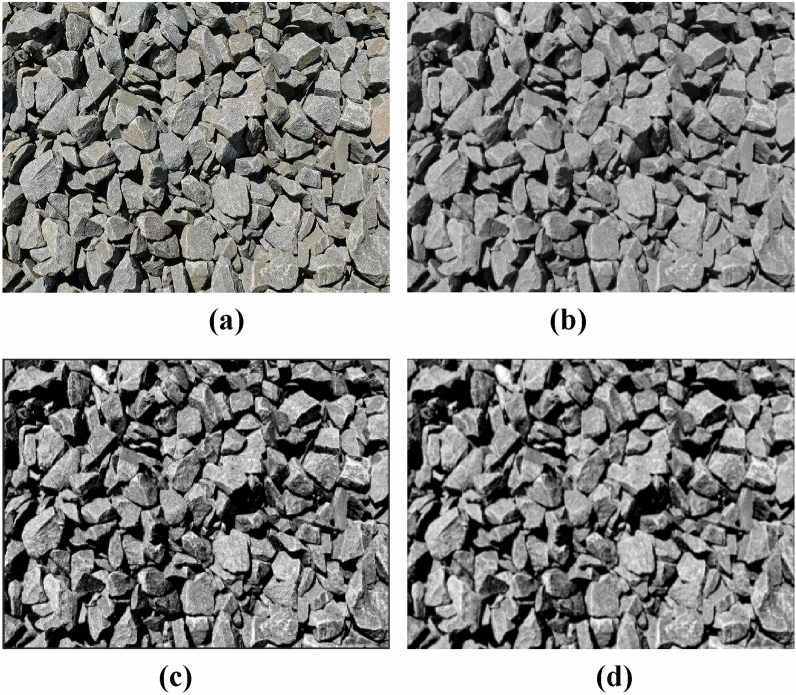
The effects of each stage of image preprocessing for blast heap. (**a**) Original image. (**b**) Grayscale transformation. (**c**) Histogram equalization. (**d**) Gaussian filtering.

Due to the complex construction site environment and the presence of significant amounts of smoke and dust, blast heap images captured by digital cameras often suffer from significant noise. The blast heaps are often highly disordered, with rocks sticking together, resulting in small background differences and subtle color information. To effectively segment the rock blocks, it is essential to preprocess the blasting rock heap image. Initially, the image undergoes grayscaling to remove color information. Then, adaptive histogram equalization is applied to enhance image contrast, revealing finer details. Finally, Gaussian filtering is utilized to remove noise while preserving edges. Figure 8 displays the original blast heap image and the effects of each stage of preprocessing.

### Improved genetic algorithm

Traditional genetic algorithms possess efficient computation capabilities, but they also exhibit the limitation of local optimization. To overcome these challenges, genetic algorithms have been enhanced, with a primary focus on optimizing the selection, crossover, and mutation operators. These enhancements aim to improve the overall performance and capability of genetic algorithms.

**Figure 9:**
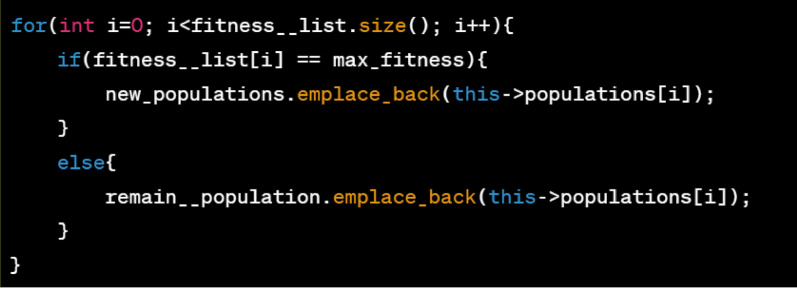
Improved genetic algorithm core code.

First, improve the roulette selection method in the genetic algorithm. In the basic roulette selection method, if a mutation produces a new maximum value (closer to the optimal value), but its population size is only 1, far less than the current maximum value (farther from the optimal value). If the roulette selection method is used directly, then this new mutated value will be overwritten. By forcing the maximum value in the previous generation to be preserved, to ensure that the population does not degenerate. The core code is shown in Fig. 9.

For genetic algorithm, the performance and convergence of the algorithm are directly affected by the crossover and mutation values. If they are set to fixed values (range 0.4–0.9), it will lead to the inability to obtain the optimal image segmentation threshold. For the crossover operator *P*_*c*_, make *P*_*c*_ two different probability values. In the early stage of iteration, to avoid the issue of searching for the global optimum caused by the eliminating of too many individuals, we increase the crossover probability value to 0.8. When in the middle and late stage, in order to enhance its convergence speed and ensure excellent individuals, the crossover probability value can be appropriately reduced (that is, it can be set to 0.6) to optimize the search for the global optimum value, and then obtain good segmentation results. For this, this study makes the following optimization, as shown in Eq. (1):

1 $$P_{c} = \left\{ \begin{array}{*{20}l} 0.8 \, gen \le 20 \hfill \\ 0.6 \, gen > 20 \hfill \\ \end{array} \right.,$$

where *gen* is the number of iterations, which makes the probability of individual selection increase in the early stage of iteration; and prevents the loss of excellent individuals in the later stage. For the mutation operator *P*_*m*_, set *P*_*m*_ to a variable probability value, as shown in Eq. (2):

2 $$P_{m} = \left\{ \begin{array}{*{20}l} 0.02 \, gen \le 30 \hfill \\ 0.03 \, gen > 30\;\& \;gen \le 50 \hfill \\ 0.02 \, gen > 50 \hfill \\ \end{array} \right..$$

**Figure 10:**
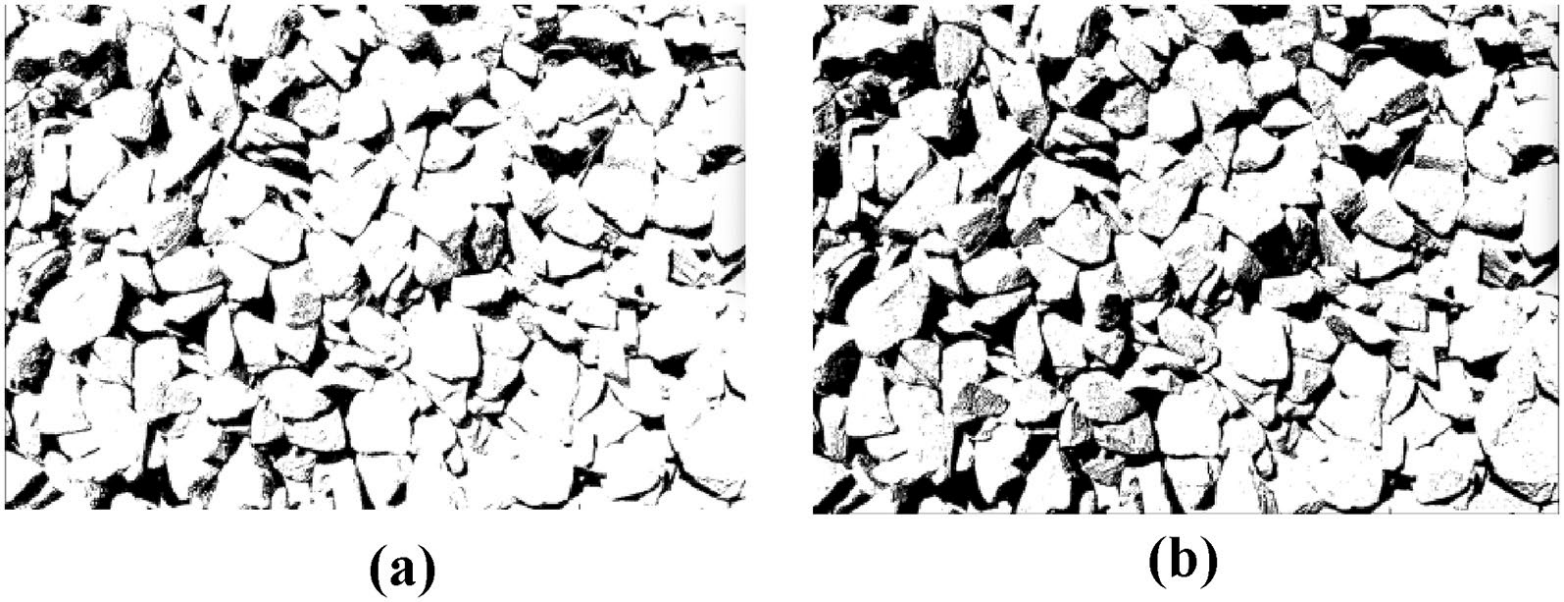
Improved genetic algorithm effect diagram. (**a**) Traditional genetic algorithms. (**b**) Improved genetic algorithms.

The image processing effects of the traditional and improved genetic algorithm are shown in Fig. 10.

### Improved Otsu algorithm

The key point of image segmentation by threshold method is the selection of the threshold *t*. If the value of *t* is too high, the background will be mistaken for the target part, which will interfere with the subsequent series of image processing operations, such as feature extraction, etc.; if the value of *t* is too low, the target area will be divided into the background area, resulting in the loss of useful information. Therefore, the selection of the optimal threshold *t* is particularly important for accurate image segmentation. As shown in Eq. (3):

3 $$\delta_{B}^{2} = b_{0} (u_{0} - u)^{2} + d_{1} (u_{1} - u)^{2} .$$

Combining the above, when the difference between the target object ${\delta }_{B}^{2}$ and the background area in Eq. (7) is more significant, the image segmentation effect is better. It can also be understood that the obtained threshold makes the two parts of the image separated from the center of the image by a large distance [i.e., *u*_0_(*t*) and* u*_1_(*t*)]. The distance between the two parts of the image can be expressed as (assuming the measure is 1 distance), as shown in Eq. (4):

4 $$d^{2} (t) = [u_{0} (t) - u_{1} (t)]^{2} .$$

The principle is the same as above, the larger the value of *d*^2^(*t*), the better the segmentation effect. Moreover, the distance between each pixel and the center in the two parts should be reduced as much as possible, indicating that the cohesion between pixels is good. Therefore, the concept of average variance is proposed, which can describe the cohesion between pixels. As shown in Eqs. (5) and (6):

5 $$\overline{\sigma }_{0}^{2} (t) = \frac{1}{{b_{0} (t)}}\sum\nolimits_{0 \le i \le t} {[i - u_{0} (t)]^{2} p(i)} ,$$

6 $$\overline{\sigma }_{1}^{2} (t) = \frac{1}{{d_{1} (t)}}\sum\nolimits_{0 \le i \le L - 1} {[i - u_{1} (t)]^{2} p(i)} .$$

The smaller the values of $\overline{\sigma }_{0}^{2}$ and $\overline{\sigma }_{1}^{2}$, the more uniform the pixel distribution of each part, the better the cohesion, and thus the better the image segmentation effect. Combining the above two factors, while maintaining a large distance between the two parts, ensure that the pixel cohesion between each part is good, so as to better segment the image. Therefore, a new optimal threshold acquisition method is derived from the Otsu basic algorithm based on the above analysis. As shown in Eq. (7):

7 $$G(t) = \frac{{b_{0} (t)d_{1} (t)d^{2} (t)}}{{\overline{\sigma }_{0}^{2} + \overline{\sigma }_{1}^{2} }} = \frac{{b_{0} (t)d_{1} (t)(u_{0} (t) - u_{1} (t))^{2} }}{{\overline{\sigma }_{0}^{2} + \overline{\sigma }_{1}^{2} }}.$$

When *G*(*t*) value is the maximum, the corresponding gray level is the best threshold *T*_*h*_. As shown in Eq. (8):

8 $$T_{h} = \arg \max [G(t)], \, t \in G_{L} .$$

**Figure 11:**
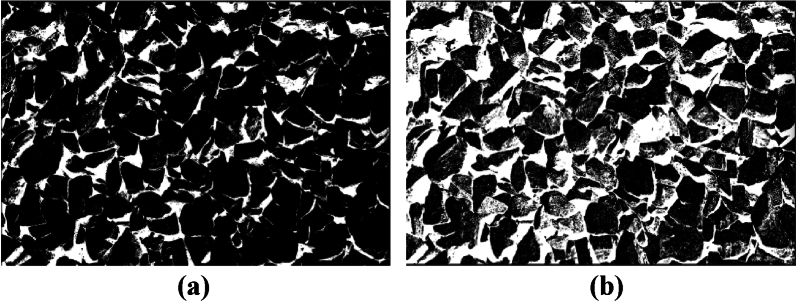
Improved Otsu algorithm effect diagram. (**a**) Traditional Otsu algorithm. (**b**) Improved Otsu algorithm.

The image processing effects of the traditional and improved Otsu algorithm are shown in Fig. 11.

### Improved genetic algorithm combined with improved Otsu algorithm

**Figure 12:**
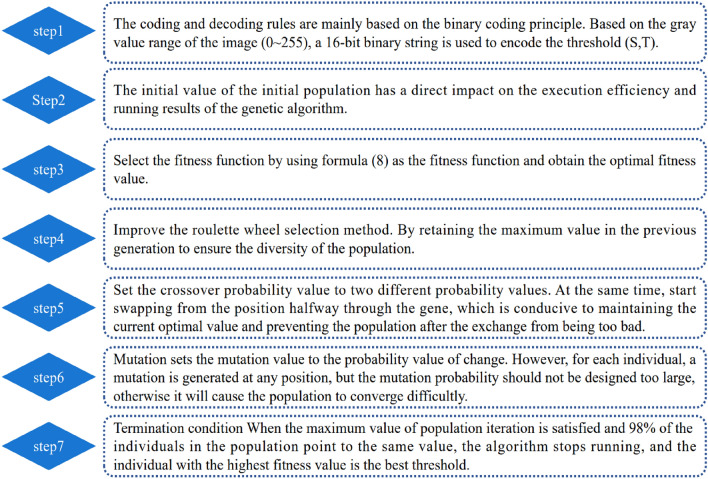
Core steps of improved genetic algorithm and improved Otsu algorithm.

The process of obtaining the optimal threshold by Otsu algorithm is equivalent to solving equation. Therefore, it can be combined with the improved genetic algorithm to improve it. The main core steps of the combination of the two algorithms are described as shown in Fig. 12.

### Marker-based watershed second segmentation

Since the image segmentation after the improved genetic algorithm maximum inter-class variance method is still not ideal for the edge segmentation of some adherent fragments in the image, it is necessary to use the marker-based watershed method to perform secondary segmentation on the threshold-segmented image to achieve better segmentation results. Before performing secondary segmentation on the image, an edge detection operator is used to enhance the edge of the obtained binary image. In order to find the edge strength and direction at position (*x*,*y*) of an image *f*, the tool chosen is the gradient, which indicates the direction of the maximum rate of change of *f* at (*x*,*y*). The Sobel operator is a first-order edge enhancement operators, which can suppress noise interference well. It uses horizontal and vertical derivatives to obtain approximate values of the gradient.

#### Horizontal change

Convolve the image *f* with an odd kernel *G*_*x*_. For example, when the kernel size is 3, as shown in Eq. (9):

9 $$G_{x} = \left[ {\begin{array}{*{20}c} { - 1} & 0 & 1 \\ { - 2} & 0 & 2 \\ { - 1} & 0 & 1 \\ \end{array} } \right] \times f.$$

#### Vertical change

Convolve the image *f* with an odd kernel *G*_*y*_. For example, when the kernel size is 3, as shown in Eq. (10):

10 $$G_{y} = \left[ {\begin{array}{*{20}c} { - 1} & { - 2} & 1 \\ 0 & 0 & 0 \\ 1 & 2 & 1 \\ \end{array} } \right] \times f.$$

For all pixels in the image *f*, combine the above two results to obtain the gradient approximation *G* as shown in Eq. (11):

11 $$G = \sqrt {G_{x}^{2} + G_{y}^{2} } .$$

**Figure 13:**
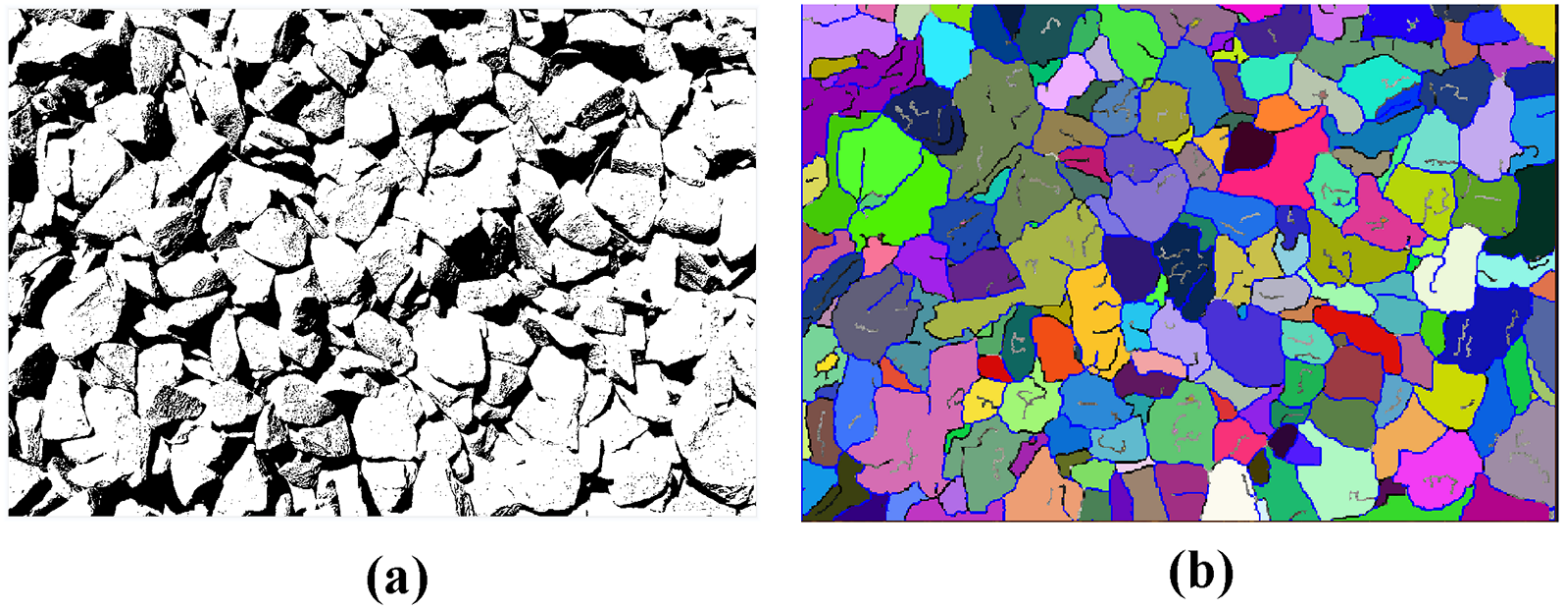
Secondary segmentation effect diagram of marker-based watershed. (**a**) Improved genetics + improved Otsu. (**b**) Improved genetic + improved Otsu + marker watershed secondary segmentation.

As shown in Fig. 13, after edge enhancement, the binary image is once again segmented using the marker-based watershed segmentation method. This method effectively segments both the adherent fragments and the edge of the image. Subsequently, morphological operations such as erosion and dilation are used separately to remove noise and smoothen the image edge.

**Figure 14:**
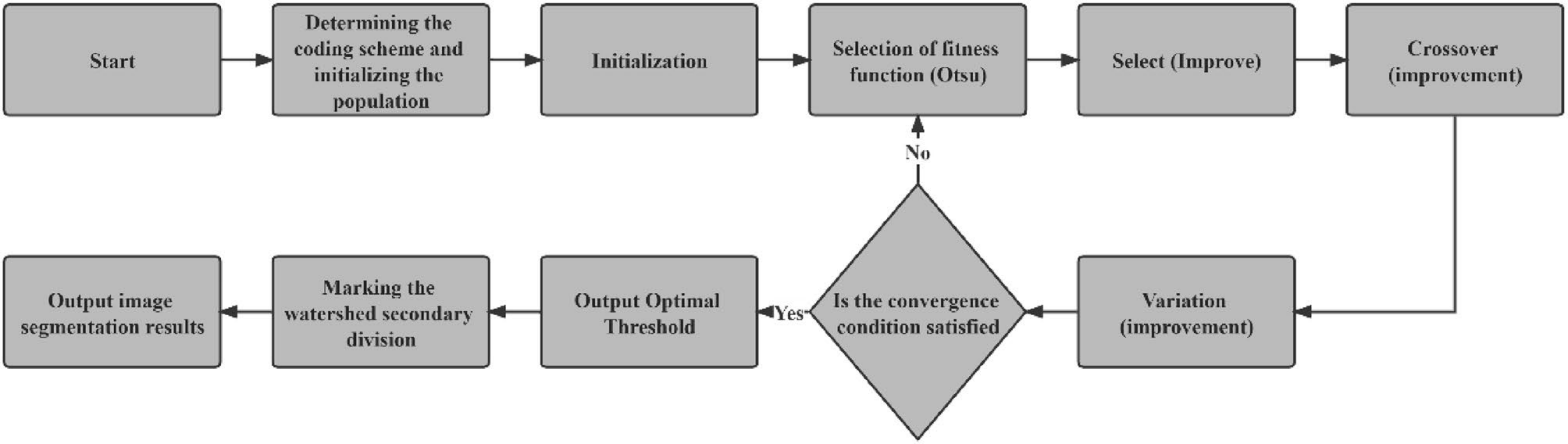
Flow chart of the image segmentation algorithm in this paper.

The overall segmentation process of this study is shown in Fig. 14.

## Blasting particle size analysis system

This study has developed a tunnel blasting fragmentation intelligent identification and efficiency dynamic feedback analysis system. This system is based on the improved Otsu algorithm, improved genetic algorithm, and marker-based watershed algorithm. It integrates the front and back modules of the platform, as well as other comprehensive systems that identify blasting particle size distribution. It records real blasting effects and analyzes gravel particle sizes at the construction site. The system quickly quantifies blasting effect feedback, reducing the need for blasting fragmentation detection technology. It also reduces personnel work intensity and impact on loading and hauling work. Overall, it enables comprehensive analysis and evaluation of the site’s blasting effect.

**Figure 15:**
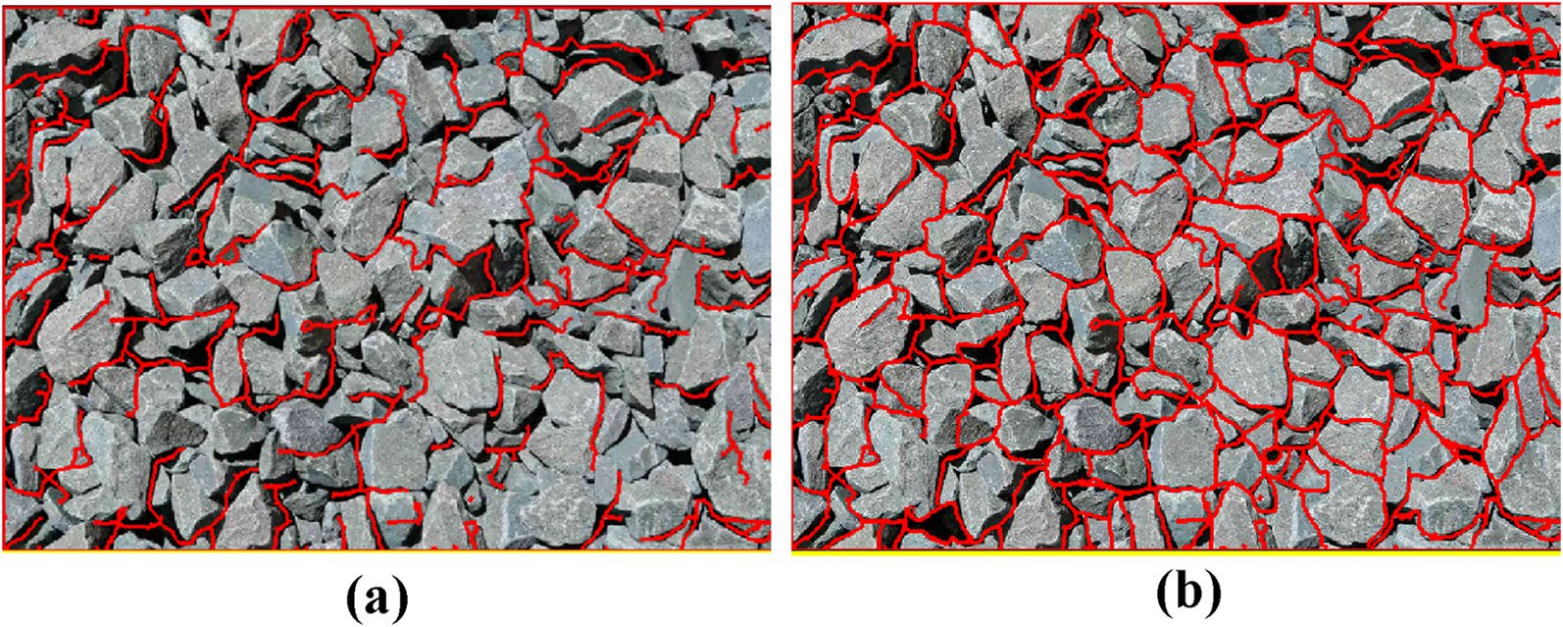
Image segmentation in different processing modes. (**a**) Quick mode. (**b**) Professional mode.

The software has the following functions:

1. It introduces the basic information of the project, such as the project location, excavation method, face rock mass grade and other basic information.
2. It grasps the construction progress, updates the tunnel construction progress according to the uploaded mileage information.
3. Image processing method. The system provides intelligent analysis and processing (fast processing) and professional processing. The difference between professional processing and fast processing in this software is that fast processing focuses on performing these operations efficiently, usually through optimization algorithms, parallel computing, or hardware acceleration. Image-specific processing is more focused on fine-grained control of image quality and features, and may employ more complex algorithms and parameter tuning to meet the requirements of specific applications. As a result, image-specific processing may sacrifice some speed to get more accurate results. The professional processing of images requires comprehensive professional knowledge in digital image foundation, filtering, edge detection, image segmentation, morphological operation, feature extraction, genetic algorithm, etc., combined with the knowledge in the field of tunnel engineering, familiar with relevant software and tools, and has the ability to optimize algorithms. The difference between the two methods is shown in Fig. 15.
4. Image interpretation. The system will extract the basic information from the processed image, which is convenient for identifying the gravel fragments, input the large fragment size specified by the site, and then get the large fragment rate after interpretation.
5. Interpretation overview. According to the system to classify the different particle sizes on the image, get the proportion of each particle size and the specific value of the large fragment rate after this blasting, and use marker-based watershed method to distinguish colors.
6. Historical data. The system will upload the records after each blasting to the cloud, recording the time, location, portal, mileage stake number and large fragment rate of each blasting. According to different time periods and mileage segments, it can also filter and quickly and accurately obtain the target information.

**Figure 16:**
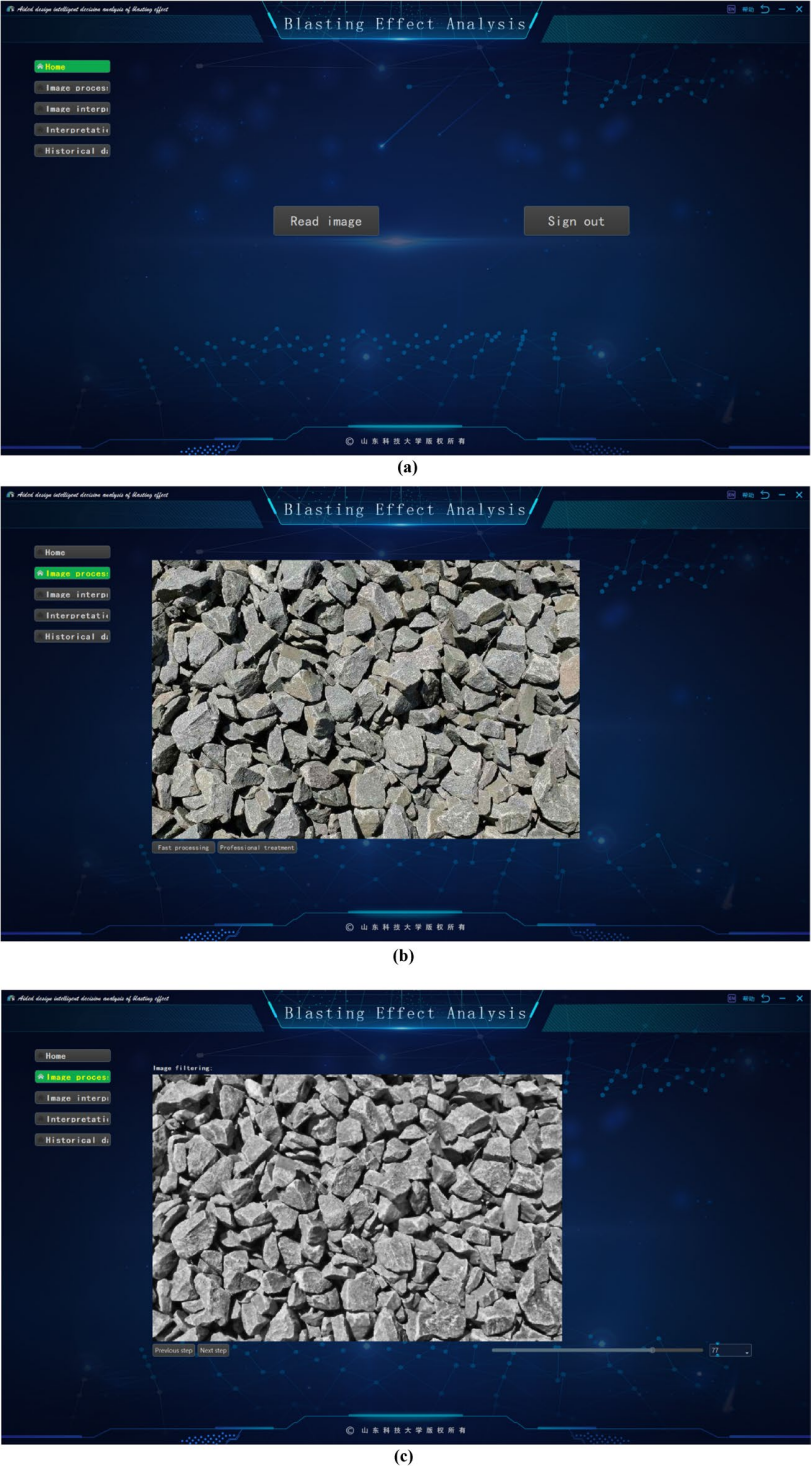

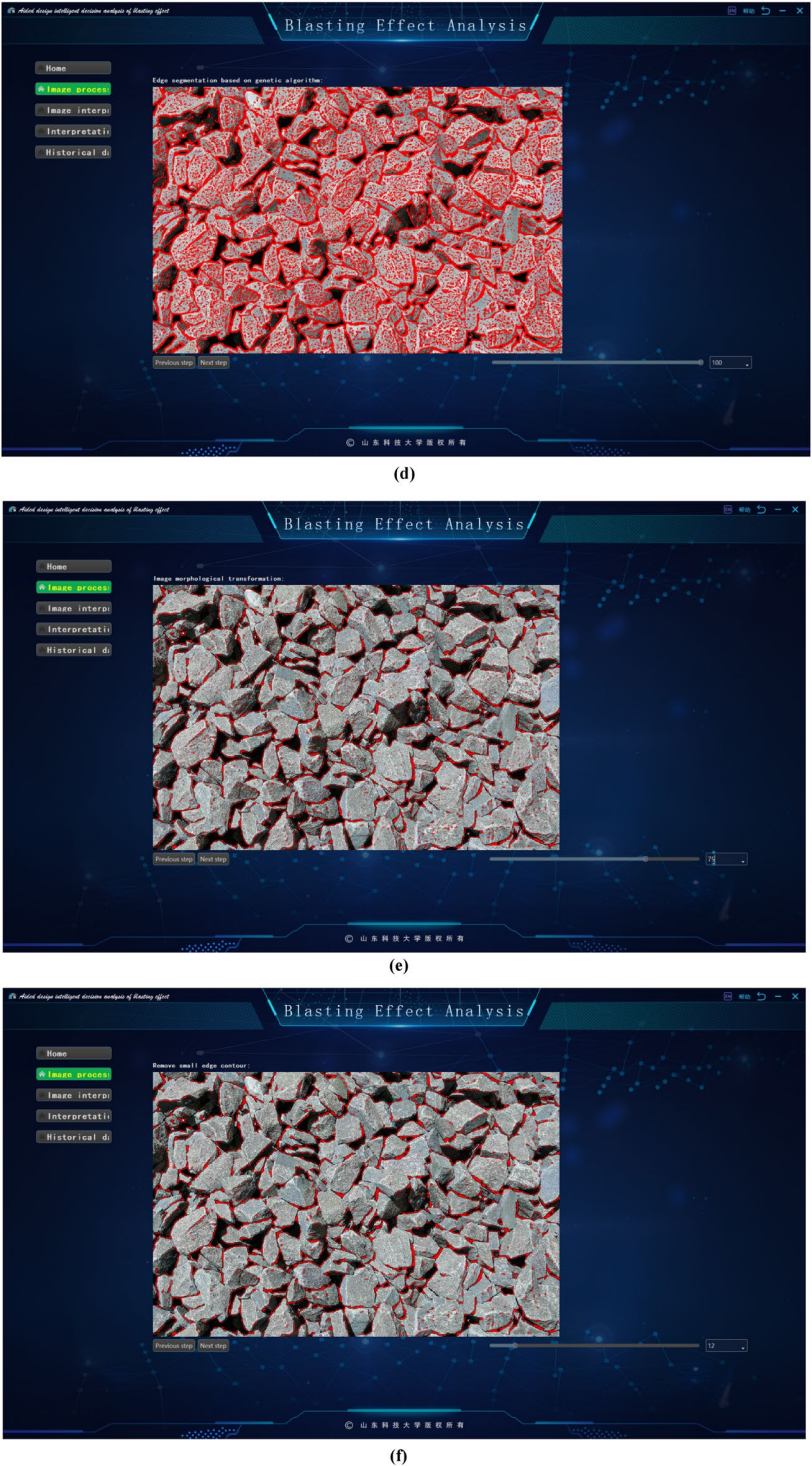

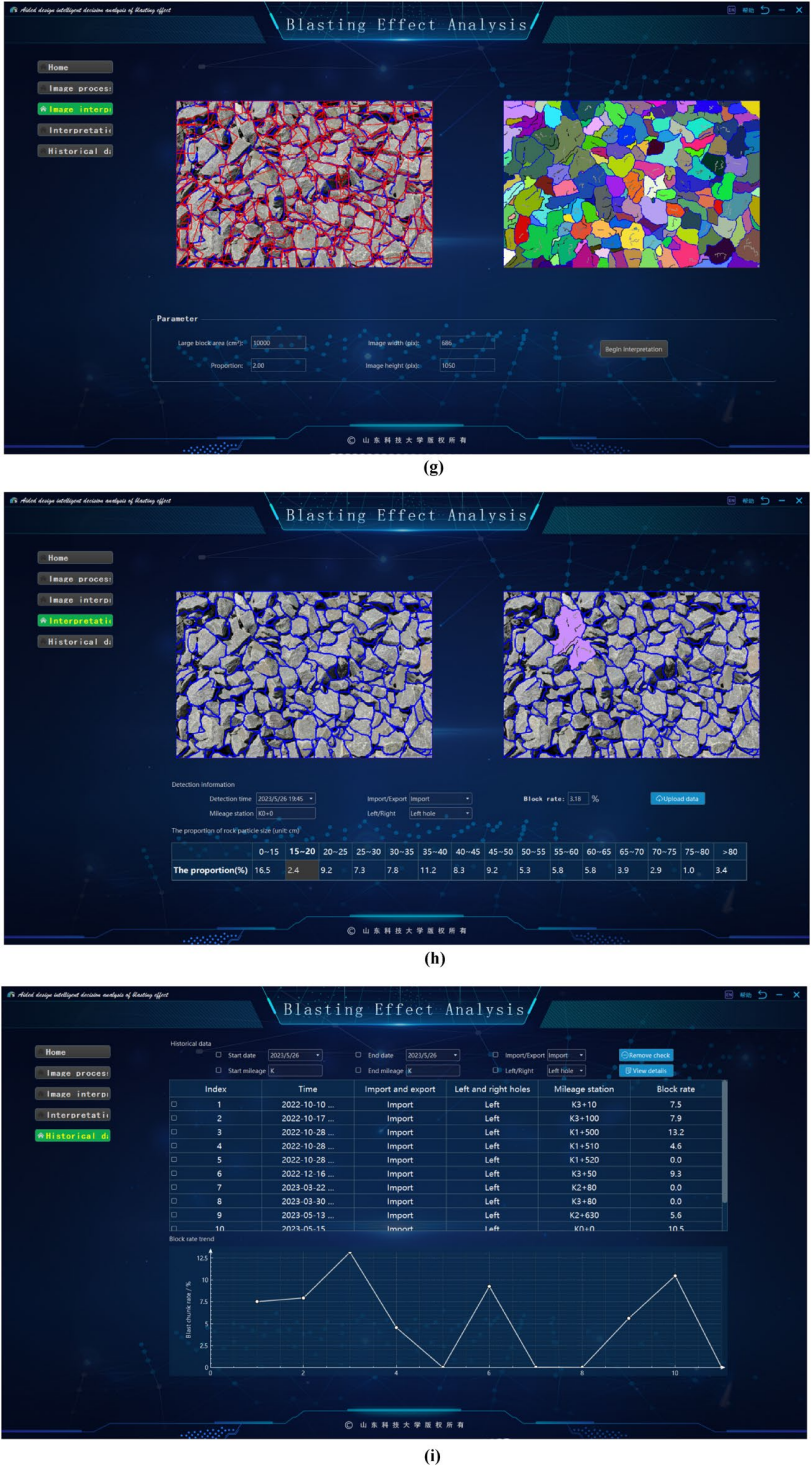

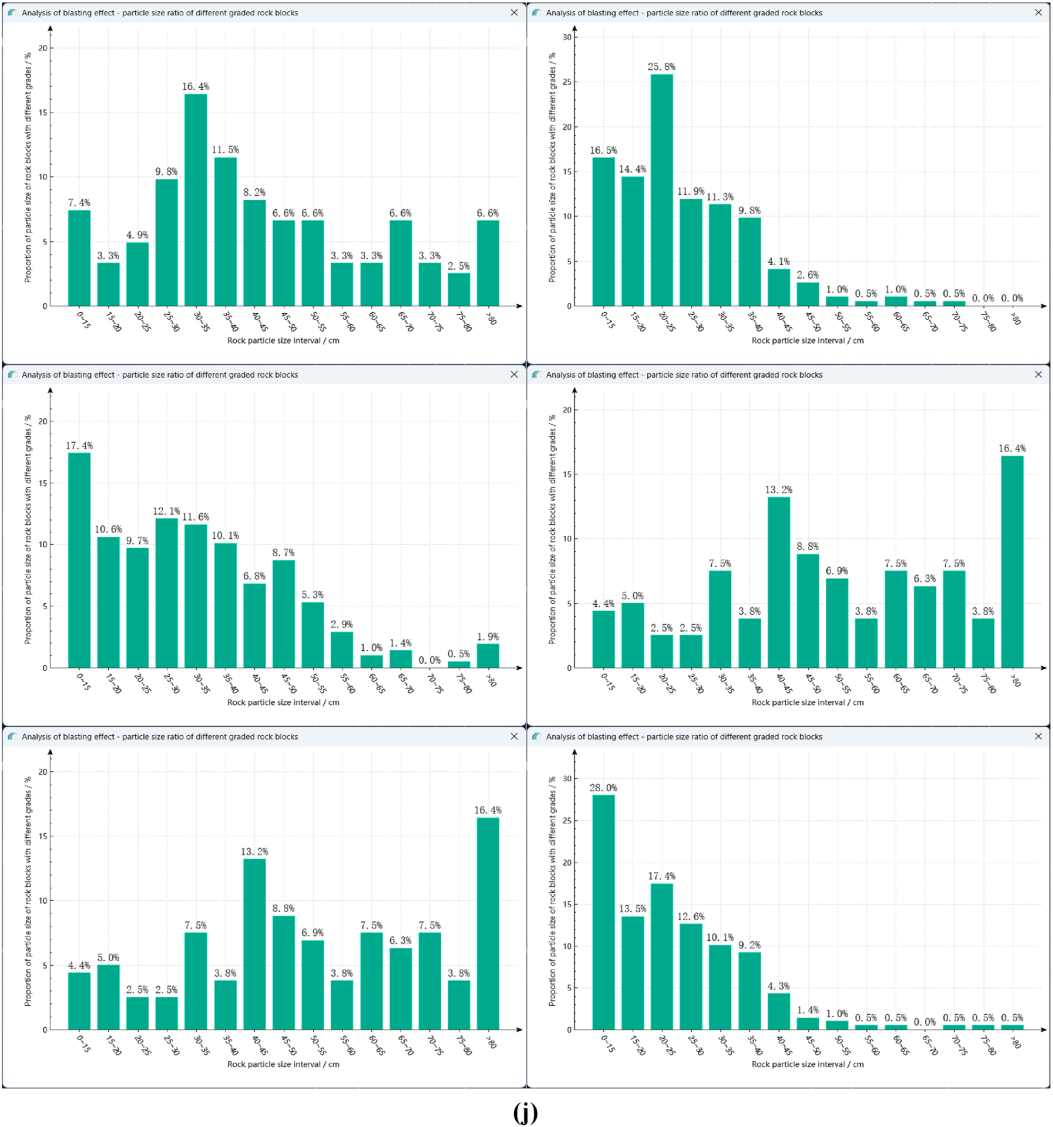
Screenshot of the software developed in this paper. (**a**) Software home. (**b**) Software processing. (**c**) Image filtering. (**d**) Genetics-based algorithm for edge segmentation. (**e**) Image morphology transformation. (**f**) Remove small edge contours. (**g**) Block degree recognition. (**h**) Chunk rate calculation. (**i**) Chunk rate cumulative curve. (**j**) Histogram of gravel particle size distribution automatically generated by the system.

This paper presents a blasting effect analysis software, which automatically generates important indicators for evaluating the blasting fragmentation effect, such as fragmentation histogram, fragmentation cumulative curve and so on, by analyzing the blasting fragmentation image. It realizes the fast detection and fragmentation identification of the blast heap, provides technical support for the optimization of blasting parameters, reduces the construction cost and improves the engineering efficiency, as shown in Fig. 16.

## Results and discussion

### Algorithmic advantages

Traditional methods sometimes choose lower, conservative thresholds for image processing in order to achieve universality for a variety of pictures, but this can cause problems such as excessive noise, which affects the subsequent analysis. And it is easy to be affected by the external environment (light, shadow, etc.), which leads to the low accuracy of these traditional image processing techniques for the identification of fissures, and it is difficult to meet the engineering needs. In contrast, compared with the Otsu algorithm in this paper, the image segmentation method based on genetic algorithm can not only effectively separate the target from the background, but also filter out a large number of images when the segmentation is fuzzy and the target is difficult to distinguish from the background. At the same time, it preserves the edges of the fragmentation and restores the original image appearance of the fragmentation as much as possible.

### Discussion of algorithm effectiveness

**Figure 17:**
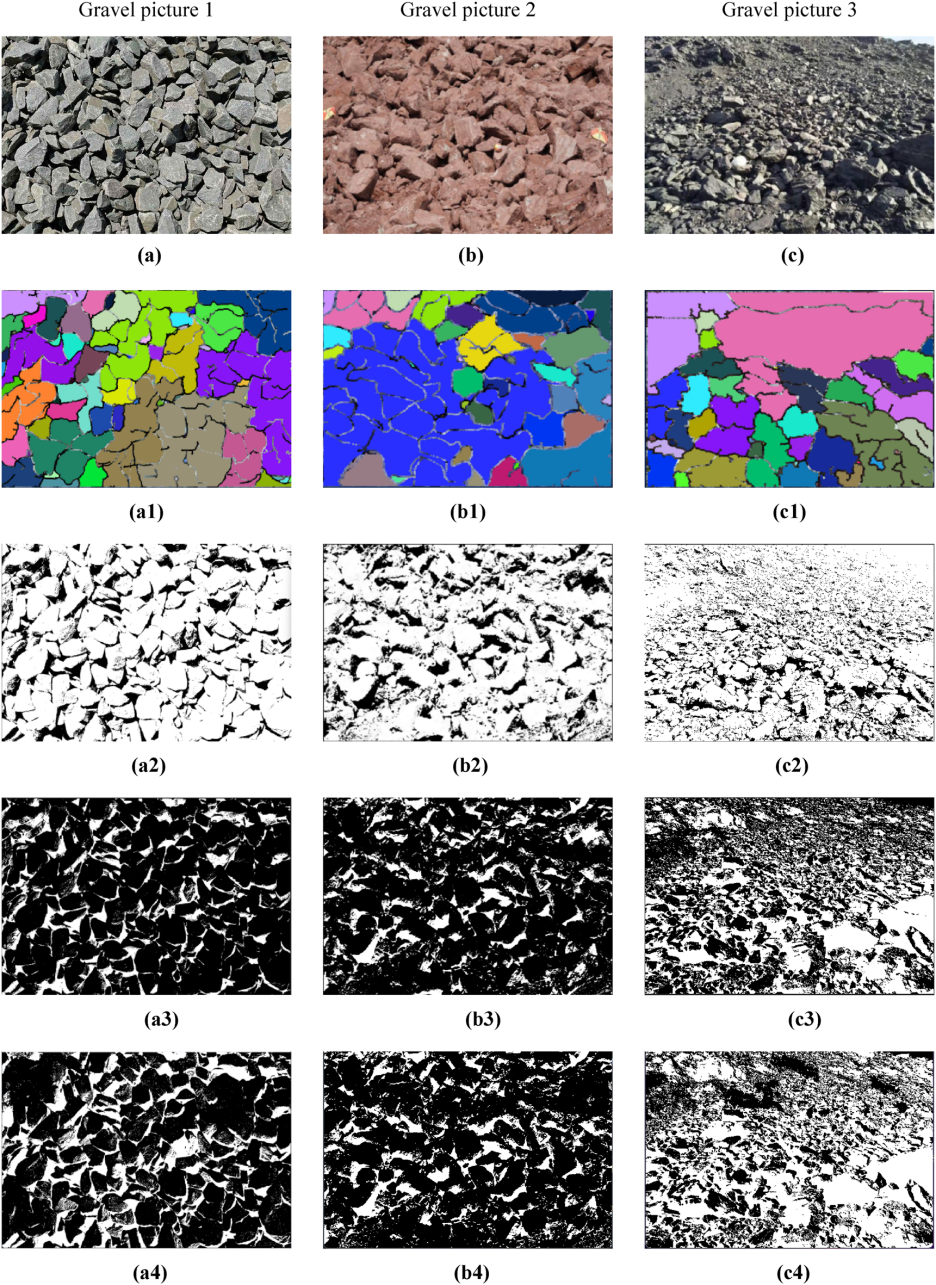

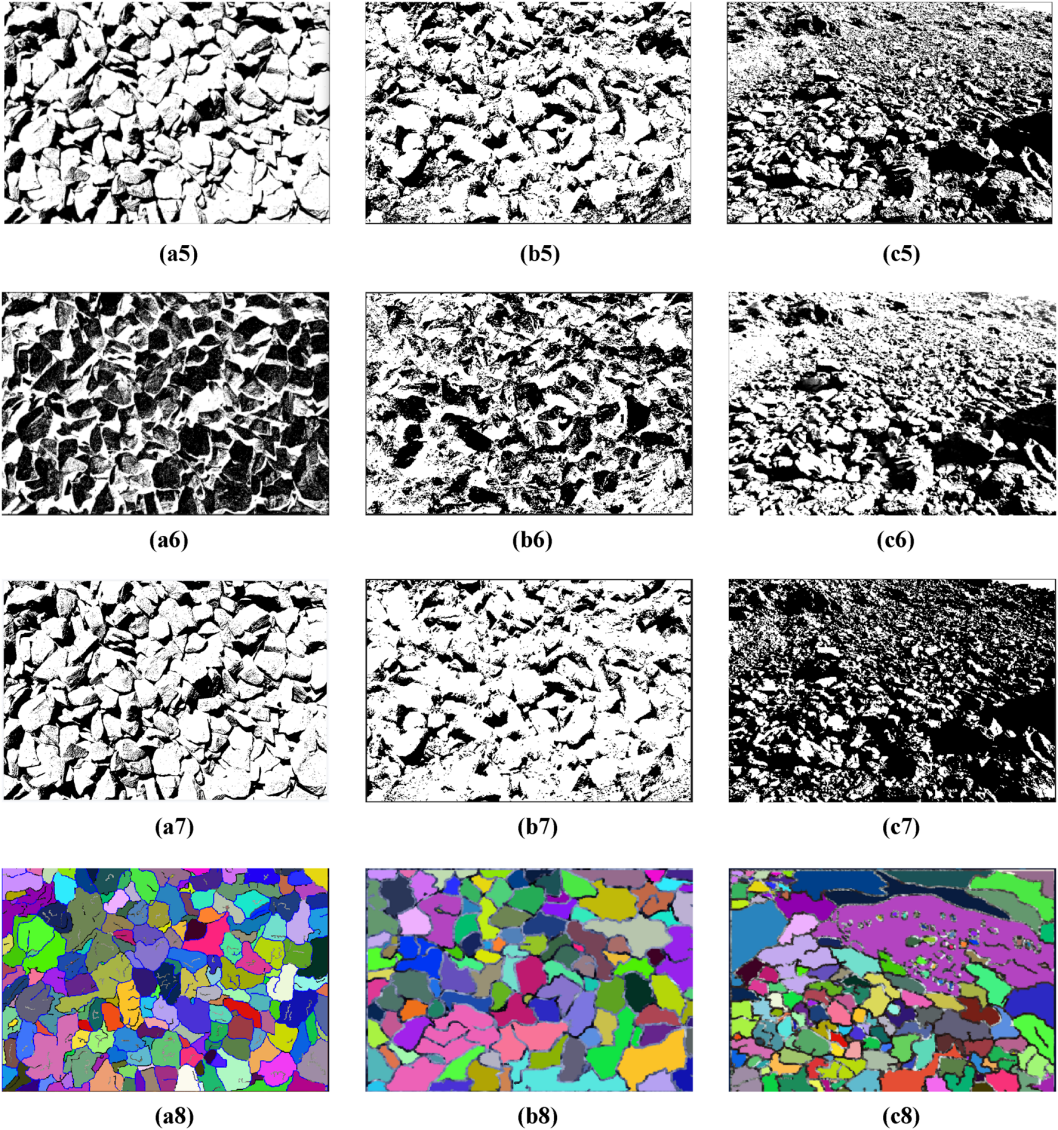
Schematic diagram of various image processing methods. (**a–c**) Original image. (**a1–c1**) Marker watershed. (**a2–c2**) Genetic algorithm. (**a3–c3**) Otsu algorithm. **(a4–c4)** Genetic algorithm + Otsu algorithm. (**a5–c5)** Improved genetic algorithm. (**a6–c6**) Improved Otsu algorithm. (**a7–c7**) Improved genetic algorithm + Improved Otsu algorithm. (**a8–c8**) Improved genetic algorithm + Improved Otsu algorithm + Marked Watershed.

The experimental environment of this study is the Windows operating system with C ++ compiler and corresponding development environment installed, processor is Intel Core i5-7400@3.00 GHz, memory is 8.0 GB. The collected blast heap images are segmented using marker-based watershed segmentation, genetic algorithm, Otsu algorithm, genetic algorithm combined with Otsu algorithm, improved genetic algorithm, improved Otsu algorithm, improved genetic algorithm combined with improved Otsu algorithm and the proposed algorithm respectively. The schematic diagram of various image processing methods are shown in Fig. 17. The specific segmentation effect of the proposed algorithm is evaluated by comparing several algorithms.

As can be seen from Fig. 17a1 using marker-based watershed segmentation alone will produce some regions where adherent fragments are not successfully segmented when facing the uneven surface and severe adhesion of the blast fragments, resulting in relatively high segmentation errors. Figure 17a2 using the traditional genetic algorithm will result in unstable segmentation results, leading to extremely poor segmentation effects; Fig. 17a4 using the genetic algorithm combined with Otsu algorithm reduces the segmentation time and improves the segmentation accuracy compared to the traditional genetic algorithm, but there are still some adherent fragments at the edge that have not been segmented. Figure 17a7 using the improved genetic algorithm combined with the improved Otsu algorithm has a better segmentation effect on adherent fragments, but there are still some fragments that have not been effectively identified, which is mainly caused by over-segmentation of the edge. Figure 17a8 using the proposed segmentation method has the best effect, not only can effectively segment the unsegmented adherent fragments in the image, but also compared to Fig. 17a5,a6 segmentation algorithms, produces the least over-segmentation phenomenon and has the most ideal segmentation effect.

In order to better quantify the segmentation effect, this paper uses the segmentation error index and evaluates the segmentation effect of the above several segmentation methods. The segmentation error is defined as shown in Eqs. (12) and (13):

12 $$P = \sum {P_{i} } ,$$

13 $$P_{i} = \frac{{\left| {N_{s} - N_{a} } \right|}}{{N_{a} }} \times 100\% .$$

In the formula: ${P}_{i}$ is the segmentation error of each particle size of the gravel; *N*_*s*_ is the number of gravel identified for each particle size; *N*_*a*_ is the number of gravel manually screened out for the corresponding particle size.

Several segmentation algorithms segmentation results quantitative table shown in Table 1. Using this paper’s algorithm for image segmentation of blasting blockiness into the genetic algorithm to accelerate the speed of threshold segmentation, so the overall time consumed relative to the single marking watershed segmentation method increases is not much, about 0.17 s; and has the best segmentation results, the segmentation results will be closer to the manual segmentation.

**Table 1:** Segmentation algorithm effect evaluation table.

Splitting algorithm	Splitting time/s	Splitting error/%
Marker watershed	0.79	50.6
Genetic algorithm	0.32	29.2
Otsu algorithm	0.44	26.7
Genetic algorithm + Otsu algorithm	0.68	20.37
Improved genetic algorithm	0.53	18.67
Improving the Otsu algorithm	0.32	17.23
Improved genetic algorithm + Improved Otsu algorithm	0.80	11.56
Methodology of this article	0.96	8.9

### Engineering applications

The high large fragment rate after blasting may bring some impacts and hidden dangers to the tunnel project, such as increasing the difficulty of excavation and cleaning, reducing the stability and safety of the tunnel, etc., so many factors need to be considered, such as blasting parameter setting, blasting technology. In the blasting design scheme, the blasting parameters can be reasonably set, such as controlling the hole spacing and diameter size, etc., to control the rock fragmentation after blasting. Using the software in this paper to obtain accurate blasting fragmentation distribution information, timely grasp the blasting effect of this construction cycle, and provide a basis for the adjustment and optimization of the blasting design scheme.

**Figure 18:**
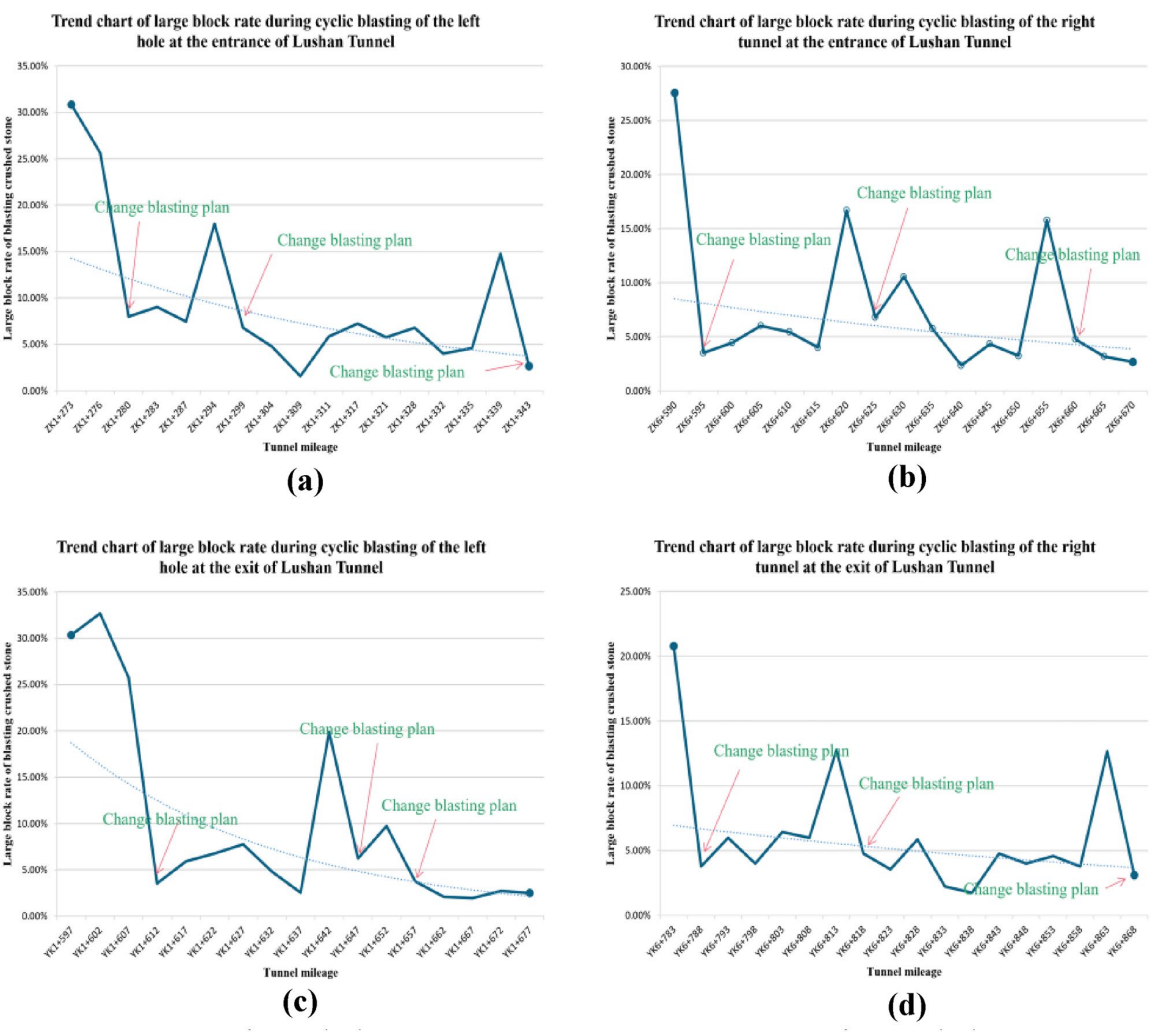
Trend charts of large fragment rates for left and right holes at the entrance and exit of Lushan tunnel. (**a**) Import left hole. (**b**) Inlet right hole. (**c**) Exit left hole. (**d**) Exit right hole.

**Figure 19:**
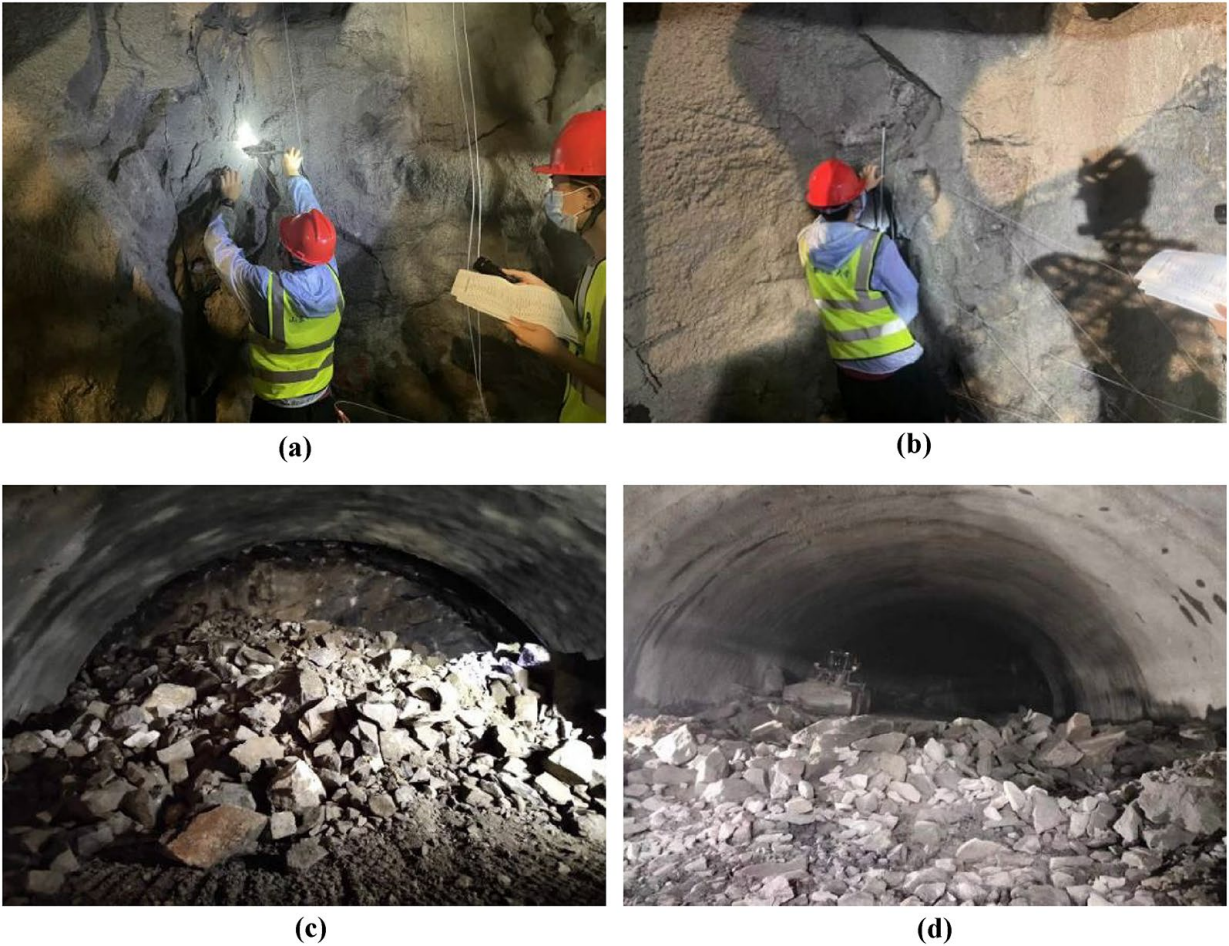
Blasting statistics of Lushan tunnel site. (**a**) Inspection and blasting program 1. (**b**) Inspection and blasting program 2. (**c**) Post-blasting debris 1. (**d**) Post-blasting debris 2.

Taking multiple blasting construction cycles of Lushan Tunnel as the research background, the blast heap images after multiple blasting operations are analyzed. It is found that the large fragment rate is high for continuous multiple blasting cycles, and some sections need secondary blasting. This not only reduces excavation efficiency but also increases cleaning difficulty. These findings suggest that the current blasting scheme for this section of the tunnel no longer meets engineering needs, necessitating a re-exploration of the site and the design of a more suitable blasting scheme based on current geological conditions. The system usage effect is shown in Fig. 18. When it is found that the current large fragment rate is too high, it indicates that the blasting effect is poor at this time, and then the blasting scheme is changed and the blasting construction parameters are optimized, so that the design of the blasting scheme is more in line with the actual engineering, improving the material utilization efficiency and reducing the engineering cost. The site pictures are shown in Fig. 19. By using the technology in this study to analyze the blasting effect, after adjusting the scheme, the blasting fragmentation is significantly reduced and the engineering efficiency is significantly improved.

## Conclusion

The research method is applied to the gravel images obtained by any low-cost sensor in the tunnel and the gravel images obtained by drone remote sensing under difficult shooting conditions. Through field tests, the algorithm proposed in this study is feasible in practical engineering, and the image information obtained by this algorithm is comprehensive, clear in details, and has practical guidance significance for engineering. It significantly improves the accuracy of image information acquisition and evaluation of gravel fragmentation after blasting, and provides data basis for the modification of blasting scheme. The following conclusions are obtained:

1. The genetic algorithm with improved genetic operators and the improved Otsu algorithm are used to perform pre-segmentation, and then the marker-based watershed segmentation algorithm is used to perform secondary segmentation on the segmented image. The experimental results show that it improves the poor segmentation effect of the traditional threshold segmentation method on adherent fragments, and reduces the over-segmentation phenomenon caused by applying the marker-based watershed algorithm alone.
2. The improved genetic algorithm speeds up the threshold segmentation, and based on the accuracy improvement, the time consumption is not much increased compared to the single marker-based watershed segmentation method, about 0.17 s.
3. The blasting effect analysis software automatically generates important indicators for evaluating the blasting fragmentation effect, such as fragmentation histogram, fragmentation cumulative curve and so on, by analyzing the blasting fragmentation image. The image recognition speed is fast and the accuracy is high.
4. The adaptive blast heap rock image recognition system based on the improved genetic algorithm developed in this paper is applied to the field blasting test, realizing the fast detection and fragmentation identification of the blast heap. By automatically generating the effect information of the blast heap, it provides technical support for the optimization of blasting parameters.

This study proposes a method based on the good application of genetic algorithm and Otsu algorithm in image segmentation, combined with marker-based watershed algorithm to improve the segmentation accuracy, and uses the software developed in this study to quickly and accurately obtain the blasting fragmentation and evaluate the blasting effect and guide the subsequent construction. However, it must be admitted that this study can only draw conclusions about whether the blasting scheme is reasonable by analyzing the blasting fragmentation. For the specific problems of the blasting scheme, further research is needed. In the future, it is possible to consider further increasing the calculation speed to ensure that it does not increase its time consumption compared to traditional methods. In order to not only quickly evaluate the blasting effect, but also propose corresponding optimization and improvement schemes.

## Data Availability

The datasets generated during and analyzed during the current study are available from the corresponding author (Feng Jiang) on reasonable request.

## References

[1] Jiang, F. *et al*. Evaluation of blasting effect based on analytic hierarchy process and cloud model in Open-pit mines. In *Proc. IEEE 3rd Int. Conf. Cloud Comput. Big Data Anal. (ICCCBDA)*, Vol. 2018, 57–61 (2018).

[2] Taji M, Ataei M, Goshtasbi K, Osanloo M (2013). ODM: A new approach for open pit mine blasting evaluation. J. Vib. Control.

[3] Yang Y (2022). Open-pit mine geological model construction and composite rock blasting optimization research. Shock Vib..

[4] Lei M, Liu L, Shi C, Tan Y, Lin Y, Wang W (2021). A novel tunnel-lining crack recognition system based on digital image technology. Tunnell. Undergr. Space Technol..

[5] Wang P, Wang S, Jierula A (2021). Automatic identification and location of tunnel lining cracks. Adv. Civil Eng..

[6] Yan, X., Zhou, G. & Zhao, X. Method for rapid detection and treatment of cracks in tunnel lining based on deep learning. In *Proc. Conference on Health Monitoring of Structural and Biological Systems IX, Electr Network*, Vol. 11381, 331–339. 10.1117/12.2558472 (2020).

[7] Huang H-W, Li Q-T, Zhang D-M (2018). Deep learning based image recognition for crack and leakage defects of metro shield tunnel. Tunnel. Undergr. Space Technol..

[8] Huang Y, Liu F, Wang J, Zhang S, Tang Q (2022). A photogrammetric system for tunnel underbreak and overbreak detection. IEEE Trans. Intell. Transp. Syst..

[9] Kemeny JM, Devgan A, Hagaman RM (1993). Analysis of rock fragmentation using digital image processing. J. Geotech. Eng..

[10] Singh BK, Mondal D, Shahid M (2019). Application of digital image analysis for monitoring the behavior of factors that control the rock fragmentation in opencast bench blasting: A case study conducted over four opencast coal mines of the Talcher Coalfields, India. J. Sustain. Mining.

[11] Yaghoobi H, Mansouri H, Farsangi MAE (2019). Determining the fragmented rock size distribution using textural feature extraction of images. Powder Technol..

[12] Hamzeloo E, Massinaei M, Mehrshad N (2014). Estimation of particle size distribution on an industrial conveyor belt using image analysis and neural networks. Powder Technol..

[13] Li XY, Huang C (2012). A novel method for image segmentation based on improved OTSU and improved genetic algorithm. Res. Explor. Lab..

[14] Purswani P, Karpyn ZT, Enab K (2020). Evaluation of image segmentation techniques for image-based rock property estimation. J. Petrol. Sci. Eng..

[15] Guo Q, Wang Y, Yang S (2022). A method of blasted rock image segmentation based on improved watershed algorithm. Sci. Rep..

[16] Bedair A (1996). Digital Image Analysis of Rock Fragmentation from Blasting.

